# Surgical Diseases of Women—Rupture of the Perineum; Its Early Management and Operative Cure

**Published:** 1864-10

**Authors:** J. F. Miner


BUFFALO

### Medical and Surgical Journal.

VOL. IV. OCTOBER, 1864. No. 3.

ART. I.—*Surgical Diseases of Women—Rupture of the Perineum; its Early Management and Operative Cure.* By J. F. Miner, M. D.

Perineal rupture is one of the most common and distressing accidents of labor, and every medical practitioner is ready to bestow upon it his best attention; few, if any, fail to meet with it in greater or less degree, in the course of their practice. There is no data, as I am aware, to show its relative frequency; the slighter degrees are certainly common, and the severer cases are more frequent than many suppose; natural modesty, and the want of any expectation that relief can be obtained, induce many sufferers to keep their malady a secret. This accident, from the nature of the case, would be more common in first labor, though it is by no means confined to primaparæ. Age may be thought also to have some influence; increased difficulty is supposed to attend first confinements after the thirtieth year, though this accident may occur in all ages. We observe different varieties according to the degrees of laceration. When the injury is slight it is of no great importance; if it extends through the recto vaginal septum, involves in rupture the sphincter ani, or even reaches the entire length of the perineum, without dividing this muscle, it constitutes the severest injury, and one which has until quite recently been generally regarded as incurable.

The various causes of laceration are sufficiently well understood; they may be comprised under two heads, the condition of the parts concerned in parturition, such as, undilated os externum, as sometimes happens in rapid labor, unyielding rigidity of perineum, structural peculiarity of the tissues, malformations of the pelvis, and the more immediately exciting causes, such as sudden and violent uterine action, great disproportion in the size of the perineum and child, improper employment of instruments, or inadequate support during the passage of the head or shoulders. It has sometimes been intimated that this accident should not occur in the hands of a careful and skillful physician, but it has occurred with the best accoucheurs and unquestionably in some cases it cannot be avoided. The best means of preventing this accident should be well settled in the mind of every practitioner. Supporting the perineum with the hand or napkin is the old and common expedient. Some authors object to this as injurious by provoking more rapid and stronger action of the uterus, but if properly conducted it is no doubt useful in directing the pressure upon the pubis, and conducting the head forward, through the external parts. It was formerly common to employ tartar emetic, warm fomentations, blood-letting, etc., but it would hardly be proposed by recent practitioners. In cases of threatened rupture, the administration of chloroform will probably do more to relax the parts and allow the safe passage of the child than all other expedients. This with sufficient and proper support would conduct many cases safely, which, without this prevention, would end in the severest rupture. When from any cause laceration is unavoidable, it has been suggested and practiced, to make during pain slight lateral incisions, thus making room for the passage of the child—producing a laceration upon the sides which could not extend into the rectum, and which would comparatively do little harm. This is a philosophical suggestion, and under proper circumstances might very well be adopted. Nature has herself in some rare instances suggested and sanctioned this procedure, lateral lacerations extending upon the side of the rectum, rather than into it, have been reported; these have terminated much more successfully, or have given much less trouble if left to themselves. Rupture during the delivery of the shoulders is quite common, and most authors sufficiently insist upon the importance of supporting the perineum during the passage of the shoulders. After the head has passed, making slight rupture, if great care is not taken, the shoulders will prolong the laceration, and greatly augment the severity of the case. The consequences of laceration depend upon the extent of rupture; many slight cases are of no great importance, while the deeper lacerations often render life miserable. It allows prolapse of the vagina, uterus and bladder, and the attendant dragging pains, incapacity for exertion, and interference with the functions of these organs, leucorrhœal discharges, and a long list of miseries which in the severer cases make life a burden.

*Treatment.*—The difficulties to be overcome spring mainly from the nature of the wound, its situation, and exposure to irritating discharges, together with the obstacles which exist in effecting apposition of the parts which shall be undisturbed for a sufficient length of time to obtain union. Discharges from the vagina, bowels and bladder, during the healing process, are liable to interfere, and the most skillful attempts have thus been frustrated. Formerly these difficulties were regarded as insurmountable, and many cases were abandoned as hopeless. There are still difficulties and uncertainties in the operation, yet they have been greatly overestimated. We may fail to obtain union even under favorable circumstances, and not unfrequently we succeed when all the opposing influences appear most active, One case, operated upon a few months since, terminated favorably, when all the common obstacles to union were augmented by the appearance, upon the third day, of most violent diarrhœa, which continued forty-eight hours, and was followed immediately by menstruation, which had come on untimely from the excitement of operation. On the other hand, cases sometimes fail when all the conditions seem most favorable, but cases which fail, or partially fail, are not to be abandoned as hopeless, another trial may prove successful; there are no obstacles to success which cannot be overcome. Influences are sufficiently numerous which may partially or wholly defeat our first efforts, but there are few, if any cases, but yield to judicious and persevering effort.

Great differences obtain among authors upon all the important points of practice, but it is not proposed to examine these conflicting opinions, but simply to express such views as appear most correct, and such as have been sustained by personal experience and observation.

What then shall be done when perineal rupture has been made—rupture which is so extensive as to demand any interference? It may be well to say that sometimes laceration, which appears to the inexperienced extensive enough to demand attention, will in a few days quite disappear, from the contraction of the parts; this is a mere hint to the young practitioner, who, very likely, will magnify the importance of the lacerations which he meets in his early practice. The smaller ruptures are often magnified in importance, while the larger ones cannot be in any way over-estimated, they constitute the sum of female woes, and their ill effects cannot be magnified.

It has been my practice of late years, when meeting with recent perineal rupture, of sufficient importance to require any attention, to introduce simple interrupted suture, one, two, or three, as the case might seem to require, and in all of my own cases, thus treated, which I now recollect, the result has been perfectly satisfactory. In one case recently treated thus at my suggestion by a medical friend, the termination is not so satisfactory and the operation for restoration is yet to be made; this plan then is certainly not always successful. In the severe forms of laceration it would not be advisable, since the quilled suture would offer much better chances of success, and even this would be uncertain in its results. Immediate restoration in extensive laceration, where the sphincter ani is divided, is much more difficult and uncertain than at a later period, when the parts are freshened anew, and there is no lochial discharge. The torn surface is not as likely to unite as the cut edges under any circumstances, and the secretions in recent cases greatly increase the liabilities of non-union. It is however well to try the plan of immediate restoration, as nothing is lost in case of failure. With this view the parts should be carefully approximated and retained for several days. Retention will be accomplished as thoroughly by the commonly described "quilled suture" as by any expedient yet suggested. Upon the proper introduction of this, will depend in great degree the chances of success. It fails from being placed too superficially, too early removed, neglect in restraining the bowels by anodynes, or of placing catheter in urethea, or of proper cleanliness by syringing, &c; it may fail from other and unavoidable causes. It has never fallen to my experience to treat for immediate restoration, a case of very extensive laceration; in the hands of careful practitioners such cases must be very rare, they do however occur from various causes, and are often presented a little later, for surgical operation. The operation is simple, safe, and usually successful, though cases of failure will sometimes occur, even when least expected. There is at times manifested no tendency in the parts to unite, an ulcerative destruction taking the place of natural adhesive inflammation.

To insure success, the freshening process should be thorough and complete; timidity and irresolution in this part of the operation is the most frequent cause of failure. The ligatures should be strong and deep and placed far back from the edge; interrupted suture may be used to nicely adjust the edges. The bowels should be quieted with morphine and a catheter retained or frequently introduced. Cleanliness and repose are also indispensable to success. It has been recommended to divide the sphincter ani muscle in order to annihilate all traction, and, in some cases, such an expedient might be useful, but it is believed to be generally quite unnecessary and to only augment the severity of the operation without greatly increasing the chances of successful termination.

It has also been recommended to relieve the tension upon the ligatures by eliptical incisions on each side of the sutured part; this can only be necessary when from any cause there has been great destruction of parts, so that there is unusual traction, in which case such an expedient might prove useful.

The deep sutures should be removed on the sixth or seventh day; if sooner, the traction might separate the recent union; if later, they are apt to produce sappurative surfaces and considerable irritation. All traction upon the parts should be avoided for a much longer period, but removal of the ligatures at this time will be proper, and greatly contribute to the comfort of the patient.

One of the most common imperfections in the results of this operation, is the continuance at some point, of a fistulous opening through which perhaps escapes the secretions from the vagina; these usually close spontaneously, or their closure may be accomplished by the application of the actual cautery, or possibly by nitrate of silver. If all this fail, the edge may be refreshened with a narrow history and a suture introduced.

Much has been written upon the subject of metalic suture for this and similar operations, and it has been claimed that the invention of the metalic suture is a discovery which ranks in importance with those of the discovery of the circulation of the blood, vaccination, application of ligature to divided vessels and anæsthesia—the most important discoveries which have ever been made in our profession so far as positive benefit to mankind is concerned. It is urged that the metalic suture induces less suppuration than any other, and that operations for vesico vaginal fistula may be successful by their use, which without would be impossible, and that nothing can really take the place of silver wire suture, where it is important to avoid suppurative action.

It does not appear why, there should be any great differences in the material of which sutures are made, only in some cases, metalic cords may be more easily fastened or adjusted than silk or linen ones, but it is not probable that waxed silk or linen, possesses any irritating qualities which are not equally common to silver or iron. When silver sutures were announced as the crowning glory of medical discovery, I commenced their use in common with others who were desirous of adopting every new and useful improvement, and sometimes I thought they were attended by less suppuration, and again I could see no manifest difference. They may be more applicable than silk or linen, and with our present plan of adjusting sutures, they have their advantages, but it is quite possible that protracted trial will finally lead to the conclusion generally that a thread of lead, iron or silver, has no advantages over one of silk or linen, where it can be as easily and nicely adjusted.

Various objections have been urged to the operation for immediate restoration. It has been supposed that acute vaginitis would be liable to occur from the effects of the sutures; that the presence of the lochia was a fatal objection; or it has been said that nature was adequate to effect a cure if left unaided. It is hardly necessary to reply to such objections; the first is a whim, not an objection; the operation of introducing sutures in cases of recent rupture is so slight that if it is not greatly magnified by unnecessary preparation, but is proceeded with unhesitatingly and unconditionally, will be completed without objection on the part of the patient; they will sometimes only regard it as a common and necessary part of the process of parturition. The lochial discharge is troublesome, but if the adjustment is perfect, and the parts are closely retained, the lochial discharge will generally produce little injury.

That nature will sometimes restore, unaided, severe lacerations is quite true, but the wonderful and exceptional manifestations of nature in restoration from disease constitute no safe and reliable basis upon which to rest from our efforts; laceration of the perineum is a malady which nature alone can sometimes cure, but nevertheless we always can, and we always should, when necessary, lend her a helping hand.

